# Selective Enhancement of REM Sleep in Male Rats through Activation of Melatonin MT_1_ Receptors Located in the Locus Ceruleus Norepinephrine Neurons

**DOI:** 10.1523/JNEUROSCI.0914-23.2024

**Published:** 2024-05-14

**Authors:** Martha López-Canul, Qianzi He, Tania Sasson, Mohamed Ettaoussi, Danilo De Gregorio, Rafael Ochoa-Sanchez, Helene Catoire, Luca Posa, Guy Rouleau, Jean Martin Beaulieu, Stefano Comai, Gabriella Gobbi

**Affiliations:** ^1^Neurobiological Psychiatry Unit, Department of Psychiatry, McGill University, Montreal, Quebec H3A 1A1, Canada; ^2^IRCCS San Raffaele Scientific Institute, Milan 20132, Italy; ^3^Department of Neurology and Neurosurgery, McGill University, Montreal, Quebec H3A 2B4, Canada; ^4^Department of Pharmacology and Toxicology, University of Toronto, Toronto, Ontario M5G 2C8, Canada; ^5^Department of Pharmaceutical and Pharmacological Sciences, University of Padua, Padua 35131, Italy; ^6^Department of Biomedical Sciences, University of Padua, Padua 35131, Italy; ^7^Research Institute, McGill University Health Center, McGill University, Montreal, Quebec H3A 1A1, Canada

**Keywords:** EEG/EMG, locus ceruleus, melatonin MT_1_ receptors, norepinephrine, rats, REM sleep

## Abstract

Sleep disorders affect millions of people around the world and have a high comorbidity with psychiatric disorders. While current hypnotics mostly increase non-rapid eye movement sleep (NREMS), drugs acting selectively on enhancing rapid eye movement sleep (REMS) are lacking. This polysomnographic study in male rats showed that the first-in-class selective melatonin MT_1_ receptor partial agonist UCM871 increases the duration of REMS without affecting that of NREMS. The REMS-promoting effects of UCM871 occurred by inhibiting, in a dose–response manner, the firing activity of the locus ceruleus (LC) norepinephrine (NE) neurons, which express MT_1_ receptors. The increase of REMS duration and the inhibition of LC-NE neuronal activity by UCM871 were abolished by MT_1_ pharmacological antagonism and by an adeno-associated viral (AAV) vector, which selectively knocked down MT_1_ receptors in the LC-NE neurons. In conclusion, MT_1_ receptor agonism inhibits LC-NE neurons and triggers REMS, thus representing a novel mechanism and target for REMS disorders and/or psychiatric disorders associated with REMS impairments.

## Significance Statement

Rapid eye movement sleep (REMS) is involved in the processes of memory consolidation and emotional regulation, but drugs selectively enhancing REMS are scant. Herein, we show that the first-in-class selective melatonin MT_1_ receptor partial agonist UCM871, by inhibiting the activity of norepinephrine neurons in the locus ceruleus, an important nucleus regulating the sleep/wake cycle, selectively increases the duration of REMS. These findings enhance our current understanding of the neurobiology and pharmacology of REMS and provide a possible novel mechanism and target for disorders associated with REMS dysfunctions.

## Introduction

Sleep disorders represent a major medical problem affecting the health and quality of life of millions of people around the world and are often in comorbidity with psychiatric disorders (Benca et al., 1992), neuropathic pain, and many other medical conditions (Taylor et al., 2007). Sleep is a succession of usually 4–6 cycles per night of non-rapid eye movement sleep (NREMS) and rapid eye movement sleep (REMS) episodes lasting ∼90 min (Atkin et al., 2018). Approved medications for sleep disorders include benzodiazepines, Z-compounds, melatonin in a prolonged-release formulation, and more recently orexin antagonists (Atkin et al., 2018). However, these drugs mostly increase NREMS and have either limited effects on enhancing REMS or suppressing REMS (Atkin et al., 2018). REMS is an essential component of sleep, which regulates not only memory consolidation and learning but also emotional processing and movement (Peever and Fuller, 2017). While several receptors activated by monoamines, GABA and glutamate are known to be involved in the regulation of REMS by acting in different and specific brain areas (Aston-Jones and Bloom, 1981; Aston-Jones et al., 2001; Atkin et al., 2018; Osorio-Forero et al., 2022), the role of the melatonin G-protein–coupled receptors (GPCRs) MT_1_ and MT_2_ is still unknown.

Polysomnography in MT_1_ receptor knock-out mice showed a selective reduction of REMS during the sleep/wake cycle, with slight impairments in NREMS, whereas the melatonin MT_2_ knock-out mice displayed a decreased duration of NREMS, with the duration of REMS not being affected (Ochoa-Sanchez et al., 2011; Comai et al., 2013). These studies suggested that MT_1_ and MT_2_ receptors may regulate REMS and NREMS differently, probably due to their unique localization within the brain areas that regulate the sleep/wake cycle (Gobbi and Comai, 2019a,b). Indeed, the pharmacological activation of MT_2_ receptors with the selective MT_2_ receptor partial agonists UCM765 and UCM924 selectively enhances the duration of NREMS without affecting REMS (Ochoa-Sanchez et al., 2011, 2014; Comai et al., 2013; Gobbi and Comai, 2019a,b), whereas preliminary findings found that the pharmacological activation of MT_1_ receptors with the selective MT_1_ receptor partial agonist UCM871 enhances REMS (Comai et al., 2017; Lopez-Canul et al., 2019). Until now, a comprehensive study of selective drugs for the MT_1_ receptor in sleep function is lacking, and the mechanism by which REMS is regulated by melatonin receptors is still unknown.

Here, we have tested in male rats the first-in-class selective melatonin MT_1_ receptor partial agonist named UCM871 (Rivara et al., 2012) and unveiled its mechanism of action in sleep. UCM871 selectively enhances REMS during the 24 h sleep/wake cycle, by decreasing, in a dose–response manner, the firing activity of the locus ceruleus (LC) norepinephrine (NE) neurons, which express MT_1_ receptors. Furthermore, using a novel adeno-associated viral (AAV) vector, we selectively knocked down MT_1_ receptors on NE-LC neurons, demonstrating that MT_1_ receptors in LC-NE neurons are essential for the MT_1_ agonism to enhance REMS.

## Materials and Methods

### Study design

The overall objective of this study was to investigate the effects of the first-in-class selective melatonin MT_1_ receptor partial agonist UCM871 on the sleep/wake cycle and to understand its mechanism of action. Using in vivo EEG/EMG in freely moving animals and single-unit extracellular recordings in anesthetized animals, we first evaluated whether the pharmacological administration of UCM871 promoted sleep and affected sleep architecture throughout the entire 24 h light/dark cycle and then according to the 12 h light/dark phases. Given the important role of the LC in the modulation of the light/dark cycle and in vigilance states (arousal and REM sleep; Aston-Jones and Bloom, 1981; Carter et al., 2010), we next decided to examine the density and distribution of the MT_1_ receptor expression in LC-NE neurons. Due to the expression of MT_1_ receptors in LC-NE neurons, we then explored the effect of UCM871 on the spontaneous neuronal firing activity of LC-NE neurons during the light phase, the phase of the day in which we observed the REMS-promoting effects of UCM871. To further demonstrate that the activity of UCM871 upon LC-NE neural firing was MT_1_ receptor-mediated, we also pretreated a group of rats with either the MT_2_ selective antagonist 4P-PDOT or the non-selective MT_1_/MT_2_ luzindole prior to the injection of UCM871. We then hypothesized that MT_1_ receptors located in LC-NE neurons were instrumental in increasing REM sleep by UCM871. After knocking down MT_1_ receptors in the LC-NE, we tested, in 24 h EEG/EMG recordings, whether the integrity of the MT_1_ receptors in LC-NE was required for UCM871 to exert its effects on REMS. Finally, in rats with knockdown of MT_1_ receptors in the LC-NE neurons, we investigated whether the electrophysiological effects induced by systemic injection of UCM871 were the result of a local effect on the LC or if they were mediated by other brain regions expressing MT_1_ receptors. In in vivo studies, the doses of UCM871 and the sample sizes used were calculated based on our previous experience with melatonin and the congener-selective melatonin MT_2_ receptor partial agonists UCM765 and UCM924 in similar experimental models (Ochoa-Sanchez et al., 2011, 2014). Rats were randomly assigned to groups before the experiments, and the number of rats per experimental group is indicated in the figure legends unless otherwise specified.

### Animals

The experiments were performed in adult male Sprague Dawley rats (225–340 g; Charles River Laboratories) housed at 22°C with *ad libitum* access to food and water and maintained under a 12 h light/dark cycle (lights on, 7:30 A.M.; lights off, 7:30 P.M.). Rats were housed in separate cages after the EEG/EMG implant. Experiments were conducted in compliance with the guidelines of and approved by the Animal Care and Welfare Committee of McGill University (protocol number 5253).

### Drugs and treatments

**Figure 1.**
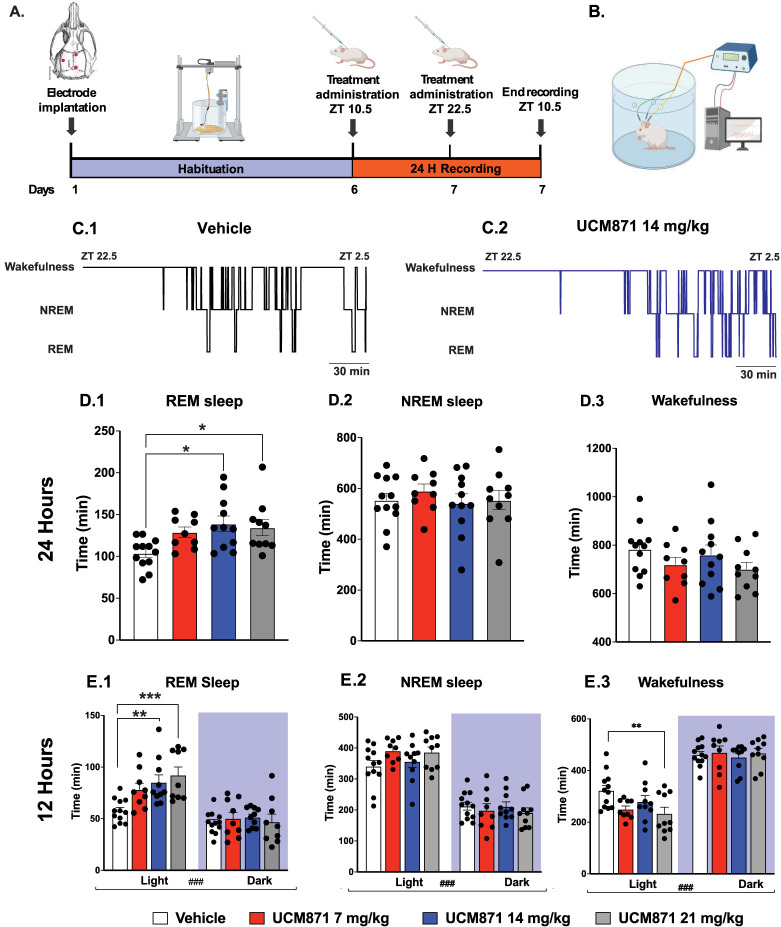
Systemic administration of the melatonin MT_1_ receptors partial agonist UCM871 increases REM sleep time during the light/inactive phase. ***A***, Schematic timeline of the experiments. ***B***, Schematic representation of the EEG/EMG system. ***C***, Examples of the duration of NREMS and REMS and wakefulness episodes during the 4 h after UCM871 administration at 6 A.M. in a rat treated with vehicle (***C.1***) and UCM871 14 mg/kg (***C.2***). ***D***, Dose–response effect of UCM871 upon the three vigilance states during the 24 h sleep/wake cycle: REMS (***D.1***), NREMS (***D.2***), and wakefulness (***D.3***). UCM871 enhances REMS during the 24 h. Four different groups of rats were used: vehicle (*n* = 12) and UCM871 at doses of 7 mg/kg (*n* = 9), 14 mg/kg (*n* = 11), and 21 mg/kg (*n* = 10), injected subcutaneously every 4 h. ***E***, UCM871 at 14 and 21 mg/kg increase REMS (***E.1***) time during the inactive/light phase. No effects of UCM871 were found on the NREMS duration (***E.2***), and UCM871 at 21 mg/kg decreased the total time of wakefulness (***E.3***). *p < 0.05, **p < 0.01, ***p < 0.001 versus vehicle; ^###^*p *< 0.001, light versus dark phase, by Bonferroni’s post hoc test.

The N-[2-[N-methyl-3-(4-phenylbutoxy)anilino]ethyl]acetamide (UCM871; https://pubchem.ncbi.nlm.nih.gov/compound/24763223; 7, 14, and 21 mg/kg; Rivara et al., 2012) was synthesized by the University of Urbino, Italy (Dr. Bedini). Luzindole (10 mg/kg) and 4P-PDOT (4-phenyl-2-propionamidotetralin; 10 mg/kg) were purchased from Sigma-Aldrich. All drugs were dissolved in a vehicle composed of 40% polyethylene glycol 400, 10% ethanol, and 50% saline. The solutions had a pH close to 5.5. The doses of 4P-PDOT and luzindole, as well as UCM871, were chosen based on our previous experiments examining the potential pharmacological activity of these compounds (Ochoa-Sanchez et al., 2014; Lopez-Canul et al., 2015a,b). Drugs were injected subcutaneously (0.5 ml, s.c.) starting at Zeitgeber time (ZT) 10.5 and every 12 h for the EEG/EMG recordings. For electrophysiological recordings, the drugs were administered intravenously between ZT 10.5 and 11.5. Figures 1*A* and 6*A* describe the experimental protocols.

### Implant of EEG/EMG electrodes, EEG/EMG recordings, and EEG/EMG data analysis

Implant of EEG/EMG electrodes and the procedures applied for EEG/EMG recording and offline analysis of the three vigilance states were performed following our previous work in rats assessing the effects of melatonin (Ochoa-Sanchez et al., 2014) or the selective MT_2_ receptor partial agonist UCM765 (Ochoa-Sanchez et al., 2011) on the sleep/wake cycle. Briefly, anesthetized rats were placed in a stereotaxic frame and implanted with three stainless-steel epidural electrodes for EEG monitoring [one over the parietal cortex on each side and the third (as a reference) in the right prefrontal cortex] and three flexible stainless-steel wire electrodes into the neck muscles for EMG monitoring (two bilaterally and one in the middle). EEG/EMG signals were recorded, subjected to a fast Fourier transform (FFT), and then analyzed offline with the Spike2 software (CED) in 10 s epochs. The three classical vigilance states were assessed based on the cortical EEG and neck EMG activities. EEG power spectra density was also computed in the frequency range of 0–64 Hz. To determine the variations in power spectra induced by the pharmacological treatment with UCM871, we summed up the power densities over the frequency band of 0–25 Hz (total power). The data were then standardized by expressing all power spectral densities at the different 0.5 Hz bin frequency ranges (δ, θ, and σ) as a percentage relative to the total power of the same epoch.

Finally, we also calculated the sleep fragmentation index (SFI) as follows: total number of awakenings from sleep (NREMS and REMS) over the total sleep time in 24 h or 12 h light phase (Morrell et al., 2000).

### Single-unit in vivo electrophysiology recording of LC-NE neurons

Rats were anesthetized with chloral hydrate (100 mg/kg, i.p.), placed in a stereotaxic apparatus (David Kopf Instruments), and a catheter was inserted into a tail vein for systemic drug administration. To maintain a full anesthetic state during the experiments, we periodically administered supplemental doses of chloral hydrate (100 mg/kg, i.p.). Anesthesia was confirmed by the absence of a nociceptive reflex reaction to a tail or a paw pinch.

Single-barrel glass micropipettes (Harvard Apparatus), filled with 2% pontamine sky blue dye (Sigma-Aldrich) dissolved in 2 M NaCl (0.5 M, pH 7.5) and with resistances ranging from 2 to 6 MΩ, were used for extracellular recording. Two burr holes with 2.5 mm diameter were drilled bilaterally at the following stereotaxic coordinates: −9.8 mm AP and 1.2 mm ML relative to the bregma (Paxinos and Watson, 2006). LC-NE neurons were recorded lowering the single-barrel glass micropipette at a depth ranging between 6.5 and 8.2 mm (Paxinos and Watson, 2006). Signals were filtered (AC, 0.2–2 kHz), amplified (Bak Electronics, model RP-I), and connected to an oscilloscope (B&K Precision; 20 MHz, model 1522) and an audio system. Signals were digitalized by a CED 1401 interface system, processed online, and analyzed offline by the Spike2 software. LC-NE neurons were identified by their firing patterns (regular firing rate, 0.5–7.5 Hz) and a broad positive action potential (0.8–1.2 ms) frequently characterized by a distinctive notch during the upward phase (Aghajanian and Vandermaelen, 1982; Labonte et al., 2012) and the transient increase of their firing rate characterized by a sequence of bursts in response to a noxious stimulus (pinch in the contralateral hindpaw; Aghajanian and Vandermaelen, 1982). LC-NE burst-firing activity, defined as a train of at least two spikes with an initial interspike interval of <80 ms and a maximum termination interspike interval of 160 ms, was also analyzed according to previously described criteria (Seager et al., 2004). Spontaneous basal firing activity of the LC-NE neurons was recorded for 5 min prior to the intravenous injection of the vehicle followed by cumulative doses of UCM871 (3.5, 7, 10.5, and 14 mg/kg), in 5 min intervals. In the antagonism experiments, luzindole (10 mg/kg, i.v.) or 4P-PDOT (10 mg/kg, i.v.) was injected 5 min after vehicle and 5 min before UCM871 administration. For the animals injected with AAV, the spontaneous firing of LC-NE neurons was assessed 25 d after the viral vector administration, and recorded for 5 min prior to the injection of vehicle or UCM871 (3.5, 7, and 10.5 mg/kg, i.v.).

Once the recordings were terminated, the pontamine sky blue dye was injected iontophoretically by passing a constant positive current of 20 μA for 10 min through the recording pipette to mark the recording site. Then rats were decapitated, and their brains extracted and stored in a freezer at −80°C. Subsequent localization of the labeled site was made by slicing 25-μm-thick brain sections using a cryostat, and the electrode placement was identified with an optical microscope (Olympus U-TV0.5XC-3). All the recording sites were within the LC according to the rat brain atlas (Paxinos and Watson, 2006).

In the vehicle groups, the firing rate was expressed as the percentage of the firing rate after vehicle injection; in the antagonist groups, the firing rate was expressed as the percentage of the firing rate after the antagonist (luzindole or 4P-PDOT) injection.

### Western blot

Non-treated rats were anesthetized with isoflurane and killed by decapitation at ZT 0.30, and their brains were immediately extracted. In addition, 300-µm-thick slices were obtained with a cryostat (Leica CM3050 S), and the LC was extracted, homogenized in RIPA buffer (50 mM Tris-HCl at pH 7.4, 150 mM NaCl, 1% NP-40, 0.1% SDS, 0.5% sodium deoxycholate, 5 mM EDTA at pH 8.0, 1 mM EGTA at pH 8.0, 10 mM NaF, 1 mM β-glycerophosphate, and 1 mM sodium orthovanadate) containing protease inhibitors (Roche), vortexed for 30 s, and centrifuged at 5,000 rpm for 5 min (Heraeus Multifuge X3, Thermo Fisher Scientific). Proteins were quantified with the Pierce BCA protein assay kit (Thermo Fisher Scientific), and samples were stored at −80°C. Proteins were resolved in RIPA buffer and 2× Laemmli buffer, heated (2 min) at 90°C, loaded in 10% SDS-PAGE (Bio-Rad), separated by electrophoresis, and transferred to nitrocellulose membranes.

The membranes were incubated (3 h) in a blocking buffer containing 5% nonfat milk in 0.1 M PBS and then rabbit polyclonal anti-MT_1_ antibodies (AMR-031, conc 1:1,000 in 5% nonfat milk in PBS-Tween 20 0.2% buffer, Alomone Labs) overnight at 4°C. To test anti-MT1A specificity, we incubated LC sections with the MT_1_ blocking peptide (catalog #BLP-MR031; conc 1:5; Alomone Labs) or without the primary Ab. The Ab recognizes the peptide (C)RVKPDNKPKLKPQD, corresponding to amino acid residues 223–236 of mouse MT_1_ (accession number Q61184) located in the third intracellular loop. Membranes were further incubated (1 h) at room temperature with horseradish peroxidase–conjugated secondary antibodies [anti-rabbit IgG (H + L), catalog #711-035-152, conc 1:20,000, Jackson ImmunoResearch]. Loading controls were performed with mouse anti–α-tubulin (catalog #MA1112, conc 1:10,000, Boster Bio) overnight at 4°C, followed by incubation (1 h) in a horseradish peroxidase–conjugated secondary antibodies [anti-mouse IgG (H + L), catalog #715-035-150, conc 1:10,000, Jackson ImmunoResearch] at room temperature. Immunoreactive bands were visualized by chemiluminescence (Bio-Rad V3 Western Workflow) and analyzed with ImageJ.

### Immunohistochemistry

To avoid possible confounding effects of the light/dark cycle on melatonin receptor immunolabeling and subcellular localization, we killed all animals between 8:00 and 10:00 A.M. Animals were deeply anesthetized with chloral hydride (100 mg/kg, i.p.) and transcardially perfused with 250 ml of 1× PBS, followed by 500 ml of 4% paraformaldehyde (PFA) in a 0.1 mM phosphate buffer. Their brains were extracted, immersed in the perfusion fixative overnight at 4°C, and transferred to a 30% sucrose solution in 1× PBS for 3 d (4°C). The brains were then immediately snapped frozen in isopentane mixed with dry ice and kept at −80°C. After, the tissue was completely embedded in an OCT compound prior to cryostat sectioning. Coronal sections of 25 µm thickness were obtained using a cryostat (Leica CM3050 S) and immersed in cryoprotective (30% ethylene glycol, 20% glycerol in PBS) solution at −20°C to preserve the tissue.

For single-labeling immunoperoxidase reactivity (*n* = 5), brain sections obtained from the cryoprotective solution were processed using a free-floating technique, thoroughly washed in 1× PBS solution, immersed in 1% hydrogen peroxide (H_2_O_2_) for 15 min, and rinsed again in 1× PBS. The sections were then immersed in 0.1% sodium borohydrate in 1× PBS for 20 min and preincubated in a blocking solution (BS) of 10% normal goat serum, 0.5% gelatin, and 0.25% Triton X-100 in 1× PBS for 2 h. For primary antibody incubation, the sections were washed in 1× PBS and incubated at 4°C with rabbit anti-MT_1_ (Alomone Labs, 1:1,000 in BS) for 48 h. Next, the sections were rinsed in 1× PBS and then incubated in biotinylated goat anti-rabbit for 2 h and then washed in 1× PBS and incubated in horseradish peroxidase–conjugated streptavidin (1:1,000 in 1× PBS) for 1 h. After rinsing in 1× PBS, the labeling was revealed with a DAB reagent kit (DAB Peroxidase Substrate Kit, catalog #SK-4100, Vector Laboratories) for 2–4 min. This reaction was quickly stopped in distilled water followed by rinsing in 1× PBS. Lastly, sections were mounted and air-dried on glass slides, dehydrated in ethanol (75, 90, 95, and 100%, 5 min each), cleared in xylene (5 min), and coverslipped with DPX (Fluka). Photomicrographs were taken at low (4×), medium (10×), and high (20×) magnifications with a Leitz Diaplan optical microscope coupled with an Olympus DP21 color digital camera and software (Olympus). Labeled cells were manually counted using ImageJ. A constant outline was used to ensure equally sized areas.

For immunofluorescent colocalization experiments (*n* = 4), the brain sections were thoroughly washed in 1× PBS and incubated in a blocking solution (BS) of PBST (0.25% Triton X-100 in 1× PBS) with 2% normal goat serum and 2% normal donkey serum for 2 h. For primary antibody binding, the sections were washed in 1× PBS and incubated at 4°C in rabbit anti-MT_1_ (Alomone Labs, 1:1,000 in BS) and chicken anti-TH (Abcam, 1:1,000 in BS) for 48 h. Following a wash in 1× PBS, the sections were incubated with Alexa Fluor 555 donkey anti-rabbit IgG secondary antibody (Invitrogen, 1:200 in BS) and goat anti-chicken IgY fluorescein conjugate secondary antibody (Invitrogen, 1:1,000) for 2 h at room temperature. Finally, the sections were rinsed in 1× PBS, mounted on gelatin-coated glass slides to air-dry, and coverslipped with Fluoroshield mounting medium with DAPI (Abcam).

*Z*-stacks of immunofluorescent images were taken under a Zeiss LSM710 confocal microscope at 20× magnification and processed with ZEISS ZEN Microscopy Software (Carl Zeiss). For quantification, at least three sections per animal were blindly analyzed by multiple operators. Quantification of positive cells was performed with the Fiji Cell Counter plugin. To visualize cells expressing AAV-hrMT_1_ (EGFP) in LC-NE neurons, we incubated the slices overnight at 4°C in chicken anti-TH (Abcam, catalog #ab76442, 1:1,000 in BS) and rabbit anti-GFP (Abcam, catalog #ab290, 1:1,000 in BS). After primary antibody incubation, the sections were washed in PBS and then incubated with a secondary antibody anti-rabbit Alexa Fluor (Invitrogen, 1:200 in BS) and anti-chicken IgY H + L (Invitrogen, 1:1,000 in BS) for 2 h at room temperature. To determine cell-specific expression levels of AAV-hrMT_1_, we performed colocalization with the semiquantitative analysis with the JACoP provided Pearson's and Mander’s overlap coefficients (http://rsb.info.nih.gov/ij/plugins/track/jacop.html) of the average of three slices per animal (*n* = 4). M1 signifies the correlation of EYFP^+^ overlapping TH^+^, while M2 indicates the overlap coefficient of TH^+^ to EYFP^+^.

### Viral vector production

#### Design of the miR-E embedded shRNA expression cassettes

Briefly, the single-stranded vector vMLC(a)-rh10 (Penn Vector Core) was produced using the custom-made AAV vector plasmid pMLC_2_. The AAV helper plasmid (Addgene #112866) for the production of the AAV serotype rh10 is described in Pillay et al. (2017). vMLC_2_-rh10 induces the constitutive expression of four different human microRNA-30-based (miR-E) (Fellmann et al., 2013) short/small hairpin (sh) RNAs directed against *Rattus norvegicus* melatonin MT_1_ receptor mRNA (NM_053676.2) and the enhanced green fluorescent protein (EGFP). Under the transcriptional control of the artificially eight times repeated cis-regulatory element PRS2 [Phox2a/Phox2b response site, identified in the human dopamine beta-hydroxylase (hDBH) promoter] combined with a minimal hDBH promoter fragment (containing a TATA box and transcription start site), it confers selective expression in noradrenergic and adrenergic neurons. Electronic sequences (TL712063VC, TL712063VA, TL712063VD, TL712063VB; OriGene) were used to create the four miR-E sequences shrMT_1_ (1–4) according to Chang et al. (2013). With up to two mismatches, no target sequences of vMLC_2_-rh10 other than that of NM_053676.2 were identified in the rat exome. pMLC2 was constructed using the GeneArt Gene Synthesis product and an AAV vector backbone provided by the facility (Neuroscience Center Zurich). The AAV vector plasmid pMLC_2_ was amplified using multiple-deletion series (MDS) strain 42 bacteria (LowMut ΔrecA; Scarab Genomics) in combination with anion exchange columns (Macherey-Nagel, NucleoBond Xtra anion exchange columns), plasmid pMLC_2_ was then characterized by Sanger DNA sequencing and restriction endonuclease analysis and dissolved in deionized and sterile water. The corresponding control AAV vector plasmid (AAVrh10/2-EGFP) was constructed identically with the difference that the short/small hairpin (sh) is nonsilencing (NS) RNAs.

#### Production, purification, and quantification of single-stranded (ss) AAV vectors

Single-stranded (ss) AAV vectors were produced and purified as previously described (Zolotukhin et al., 1999; Paterna et al., 2004). Briefly, human embryonic kidney (HEK) 293 cells (Graham et al., 1977) expressing the simian virus (SV) large T-antigen (293T; DuBridge et al., 1987) were transfected by polyethyleneimine (PEI)-mediated cotransfection of AAV vector plasmids (providing the to-be packaged AAV vector genome), the AAV helper plasmid pAAV2/rh10 (Addgene #112866, providing the AAV serotype 2 rep proteins and the cap proteins of AAVrh10), and the adenovirus (AV) helper plasmids pBS-E2A-VA-E4 (providing the AV helper functions) in a 1:1:1 molar ratio.

At 120–168 h post-transfection, HEK 293T cells were collected and separated from their supernatant by low-speed centrifugation. AAV vectors released into the supernatant were PEG-precipitated between 24 and 48 h at 4°C by adding a solution of polyethylene glycol 8000 (8% v/v in 0.5 M NaCl), followed by low-speed centrifugation (1 h at 3,500 × *g*/4°C). The cleared supernatant was discarded, and the pelleted AAV vectors were resuspended in an AAV resuspension buffer (150 mM NaCl, 50 mM Tris-HCl, pH 8.5). HEK 293T cells were resuspended in AAV resuspension buffer and lysed by Bertin's Precellys Evolution homogenizer in combination with 7 ml soft tissue homogenizing CK14 tubes (Bertin). The crude cell lysate was DENARASE (c-LEcta GmbH) treated (150 U/ml, 90–120 min at 37°C) and cleared by centrifugation (10–30 min at 7.164 × *g*/4°C). The PEG-precipitated AAV vectors were combined with the cleared cell lysate and subjected to discontinuous density iodixanol (OptiPrep, Axis Shield) gradient (isopycnic) ultracentrifugation (2 h 15 min at 365,929 × *g*/15°C). Subsequently, the iodixanol was removed from the AAV vector containing the fraction by three rounds of diafiltration using Vivaspin 20 ultrafiltration devices (100,000 MWCO, PES membrane, Sartorius) and 1× phosphate-buffered saline (PBS) supplemented with 1 mM MgCl_2_ and 2.5 mM KCl according to the manufacturer's instructions. The AAV vectors were stored aliquoted at −80°C.

Encapsidated viral vector genomes (vg) were quantified using the Qubit 3.0 fluorometer in combination with the Qubit dsDNA HS Assay Kit (Life Technologies). Briefly, 5 µl of undiluted (or 1:10 diluted) AAV vectors were prepared in duplicate. Untreated and heat-denaturated (5 min at 95°C) samples were quantified according to the manufacturer's instructions. Intraviral (encapsidated) vector genome concentrations (vg/ml) were calculated by subtracting the extraviral (nonencapsidated; untreated sample) from the total intra- and extraviral (encapsidated and nonencapsidated; heat-denatured sample).

The identity of encapsidated genomes was verified and confirmed by Sanger DNA sequencing of amplicons produced from genomic AAV vector DNA templates (identity check).

Titers of AAV shr MT_1_ vectors, as determined by quantitative PCR, were over 1 × 10^E12^ viral genomes (vg)/ml. Single-use aliquots of AAVshr MT_1_ and scrambled were stored at −80°C until surgery.

#### Viral vector administration

AAV injections (shrMT_1_ and EGFP) were made in adult rats (200–250 g) anesthetized with 2–5% isofluorane and immobilized on a stereotaxic frame (David Kopf Instruments); two burr holes with 1 mm diameter were drilled bilaterally. A single craniotomy was performed and a Hamilton syringe fitted with a 28-gauge needle was used to place a viral bolus (1 µl) at the following coordinates: 9.8 mm AP, 1.2/−1.2 mm ML, relative to the bregma, and −7 mm DV (Paxinos and Watson, 2006). The needle was connected to the drug-filled Hamilton syringe and was left in place for 5 min postinfusion to allow for diffusion of the virus away from the injection site. Next, 1 µl of viral construct or control vector was infused manually at a rate of 0.2 µl/min, followed by 5 min rest period to prevent backflow. Animals were allowed a recovery period of 26 d before use in experiments. Animals with the AAV injection were separated into two different groups: one for electrophysiology and the other implanted with EEG/EMG electrodes, for the posterior sleep/wake cycle analysis.

#### Quantitative RT-PCR

To validate the effect of the viral vector, we performed quantitative RT-PCR in shr MT_1_ (*n* = 5) and in EGFP (*n* = 5) injected animals. For this, both LC from each animal were collected between ZT 0.5 and ZT 2.5 and pulled together, and total RNA was extracted from the tissue using the RNA isolation kit RNeasy Lipid Tissue (#1023539, Qiagen) according to the manufacturer's instructions. RNA concentration and purity were determined by UV spectroscopy at 260 and 280 nm. Single-stranded cDNA synthesis was performed from 800 ng of total RNA using the SuperScript VILO cDNA Synthesis Kit (#11754050, Invitrogen) following the manufacturer's instructions. Quantitative real-time PCR was performed using the TaqMan method (Applied Biosystems) with TaqMan Fast Advanced Master Mix (#4444556, Applied Biosystems) and a combination of primers and probes MT_1_ #4331182; β-actin (ACTB) #4331182; and POLR2A #4331182 (Thermo Fisher Scientific). Real-time PCR was performed on the QuantStudio 7 Flex system for 40 cycles at 95 and 60°C. The level of expression was determined by converting the cycle threshold (Ct) values using the 2^−ΔΔCt^ method [with ΔΔCt = ΔCt (mRNA test) – ΔCt (mRNA control AAVrh10/2-EGFP)] to express the relative quantification of each mRNA level in comparison with the level of control AAVrh10/2-EGFP mRNA.

#### Pharmacokinetic (PK) studies of UCM871

PK studies were conducted measuring the levels of UCM871 in the plasma of the rats after the subcutaneous injection of 7 mg/kg UCM871 dissolved in a vehicle composed of 40% polyethylene glycol 400, 10% ethanol, and 50% saline. The following time points were assessed: 30, 60, 120, and 240 min. Three rats per time point were examined. The pharmacokinetics of UCM871 were described using a noncompartmental model (PK Solver 2.0). The analyses were performed on a TSQ Quantum Ultra triple quadrupole from Thermo Fisher Scientific coupled to a Surveyor series LC stack (Thermo Fisher Scientific) using UCM765 (Ochoa-Sanchez et al., 2011) as internal standard dissolved in LC-MS grade acetonitrile (Thermo Fisher Scientific). The column used was a Kinetex C18, 2.1 × 50 mm, 2 mm (Phenomenex). The eluents were Milli-Q water containing 0.1% formic acid and acetonitrile. The gradient started with 20% organic up to 95% in 1.5 min, held at 95% for 1 min for a total run time of 5 min. The flow was set at 0.350 ml/min and the injection volume at 2 µl. Mass spectra were acquired by electrospray in positive mode using multiple reaction monitoring (MRM) for each compound (UCM871 and UCM765).

#### UCM871 determination in rat plasma

An aliquot of 30 µl of plasma was protein precipitated with 300 µl of internal standard solution on ice. The mixture was then vortexed for 1 min and centrifuged at 4,000 rpm for 10 min at 4°C. Next, 80 µl of supernatant was mixed with 160 µl of water containing 0.1% formic acid. The mixture was vortexed and submitted to LC-MS/MS analysis.

### Statistical analysis

Data analysis was performed using Prism 8 (GraphPad) software. Data were tested for normality using the D’Agostino–Pearson’s test. When examining the effect of UCM871 on the 24 h sleep/wake parameters and electrophysiology, one-way ANOVA for repeated measures was performed. For light/dark experiments, two-way ANOVA or two-way ANOVA for repeated measures was applied. Two-tailed paired *t* test was used for immunoreactivity and PCR experiments and to assess the effects of viral vector infection. Post hoc analyses were performed using the Bonferroni’s comparisons. All data are expressed as mean ± SEM. *p *< 0.05 was considered significant.

## Results

### Pharmacokinetic properties of UCM871

Using an LC-MS/MS protocol, we determined the concentration of UCM871 in the plasma of three rats after 30, 60, 120, and 240 min (7 mg/kg, s.c.). Thirty minutes after administration, the average plasma concentration of the drug was 132.00 ng/ml, at 60 min it increased to 169.07 ng/ml, at 120 min it decreased to 88.95 ng/ml, and at 240 min it further decreased to 85.45 ng/ml. Using the PK Solver 2.0 software and a non-compartmental model, we found that the half-life of UCM871 was 210.2 min and its Cmax was 169.07 ng/ml.

### UCM871 selectively increases the duration of REMS during the 24 h light/dark cycle

Figure 1*D*.1–*D*.3 illustrates the dose–response effects of UCM871 upon the duration of REMS and NREMS and wakefulness during the 24 h light/dark cycle, respectively. UCM871 increased the duration of REMS (Fig. 1*D*.1; *F*_3,38 _= 4.45, *p *= 0.009, one-way ANOVA) at a dose of 14 mg/kg (*p* = 0.02) and 21 mg/kg (*p *< 0.006) compared with the vehicle. No significant effects of UCM871 on the duration of NREMS (Fig. 1*D*.2; *F*_3,38 _= 0.37, *p *= 0.78, one-way ANOVA) and wakefulness (Fig. 1*D*.3; *F*_3,38 _= 1.30, *p *= 0.28, one-way ANOVA) were observed.

### UCM871 selectively increases the duration of REMS during the light/inactive phase

The effects of UCM871 on the duration of REMS were phase of the day-dependent. Indeed, we found a dose-dependent effect of UCM871 on the duration of REMS in the light/inactive phase only (interaction, *F*_3,38 _= 4.22, *p *= 0.01; treatment, *F*_3,38 _= 5.22; *p *= 0.004; phase of the day, *F*_1,38 _= 54.88, *p *< 0.0001, two-way ANOVA for repeated measures). UCM871, compared with vehicle, at the doses of 14 mg/kg (*p* = 0.006) and 21 mg/kg (*p* = 0.0001) increased the duration of REMS during the light/inactive phase (Fig. 1*E*.1). The duration of NREMS was globally higher during the light phase than during the dark phase (Fig. 1*E*.2; interaction, *F*_3,38 _= 2.71, *p *= 0.06; treatment, *F*_3,38 _= 0.54, *p *= 0.65; phase of the day, *F*_1,38 _= 269.4; *p *< 0.0001, two-way ANOVA for repeated measures). Animals were more awake during the dark than during the light phase (Fig. 1*E*.3; interaction, *F*_3,38 _= 3.42, *p *= 0.027; treatment, *F*_3,38 _= 1.31, *p *= 0.29; phase of the day, *F*_3,38 _= 251.0, *p *< 0.0001, two-way ANOVA for repeated measures). A shorter duration of wakefulness during the light phase in rats treated with 21 mg/kg UCM871 compared with those receiving vehicle (*p *= 0.007) was found. As shown in Table 1, no significant changes induced by UCM871 were found for the duration (REMS, interaction, *F*_3,36 _= 0.76, *p *= 0.526; treatment, *F*_3,36 _= 2.01; *p *= 0.130; phase of the day, *F*_1,36 _= 3.26, *p* = 0.079; NREMS, interaction, *F*_3,36 _= 0.75, *p *= 0.525; treatment, *F*_3,36 _= 1.21; *p *= 0.320; phase of the day, *F*_1,36 _= 28.76, *p *< 0.0001; wakefulness, interaction, *F*_3,36 _= 0.39, *p *= 0.762; treatment, *F*_3,36 _= 1.99; *p *= 0.133; phase of the day, *F*_1,36 _= 22.69, *p *< 0.0001, two-way ANOVA for repeated measures) and the number (REMS, interaction, *F*_3,38 _= 1.24, *p *= 0.309; treatment, *F*_3,38 _= 0.91; *p *= 0.446; phase of the day, *F*_1,38 _= 20.32, *p *< 0.0001; NREMS, interaction, *F*_3,38 _= 0.08, *p *= 0.970; treatment, *F*_3,38 _= 1.70; *p *= 0.183; phase of the day, *F*_1,38 _= 54.43, *p *< 0.0001; wakefulness, interaction, *F*_3,38 _= 0.21, *p *= 0.888; treatment, *F*_3,38 _= 2.32; *p *= 0.091; phase of the day, *F*_1,38 _= 12.40, *p* = 0.0011, two-way ANOVA for repeated measures) of REMS, NREMS, and wakefulness bouts during both the light and the dark phases. However, there was a general trend for increased duration and number of REMS bouts with the three doses of UCM871 during the light phase, when the drug significantly increases REMS duration. As expected, longer duration and higher number of REMS and NREMS bouts as well as shorter duration and lower number of bouts of wakefulness were found during the light than during the dark phase. Finally, to calculate the restlessness during sleep, we calculated the sleep fragmentation index (SFI). One-way ANOVA for SFI during the 24 h exhibited no difference between groups (*F*_3,38 _= 11.808, *p* = 0.1621). Moreover, the treatment with UCM871 did not affect the SFI also during the light phase (*F*_3,38 _= 1.819, *p* = 0.1601). Altogether these data suggest that sleep architecture was intact.

**Table 1.** Number and duration (min) of REMS, NREMS, and wakefulness bouts during the 12 h light/dark phases after the subcutaneous treatment with UCM871 at the doses of 7, 14, and 21 mg/kg

		REMS		NREMS		Wakefulness	
		Light	Dark	Light	Dark	Light	Dark
Veh	Number (#)	58.1 ± 2.1	49.9 ± 4.2	153.1 ± 8.0	111.4 ± 4.9	126.1 ± 9.6	106.5 ± 5.6
	Duration (min)	1.0 ± 0.1	1.0 ± 0.1	2.3 ± 0.1	1.9 ± 0.1	2.5 ± 0.38	4.3 ± 0.4
7 mg/kg	Number (#)	70.6 ± 4.9	55.1 ± 6.3	152.0 ± 7.9	115.3 ± 8.4	125.1 ± 9.1	108.6 ± 6.1
	Duration (min)	1.2 ± 0.1	1.0 ± 0.1	2.3 ± 0.3	1.8 ± 0.24	2.3 ± 0.3	4.3 ± 0.6
14 mg/kg	Number (#)	59.1 ± 7.0	42.6 ± 6.0	131.1 ± 16.6	101.7 ± 10.3	102.1 ± 9.2	96.1 ± 9.6
	Duration (min)	1.5 ± 0.2	1.4 ± 0.1	2.6 ± 0.2	2.0 ± 0.3	2.8 ± 0.4	4.8 ± 0.7
21 mg/kg	Number (#)	72.2 ± 7.5	44.1 ± 11.0	135.2 ± 6.0	97.6 ± 5.8	131.3 ± 4.9	112.8 ± 5.4
	Duration (min)	1.3 ± 0.2	1.2 ± 0.1	2.8 ± 0.2	2.2 ± 0.1	1.9 ± 0.6	4.0 ± 0.5

Data are reported as mean ± SEM.

### UCM871 affects the power density of REMS and NREMS

**Figure 2.**
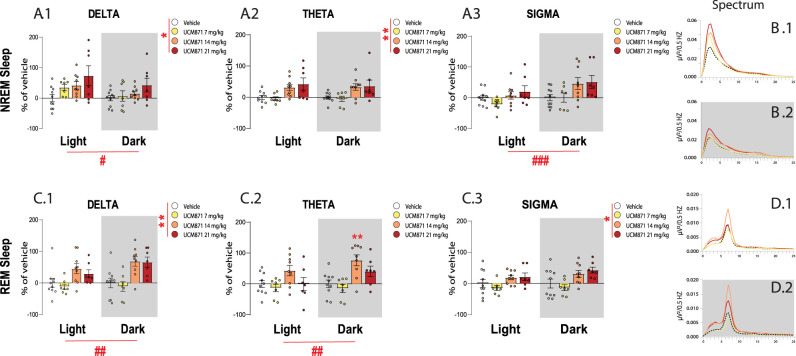
Effects of the selective MT_1_ partial agonist UCM871 on the EEG power of δ, θ, and σ bands during light and dark phase. Power spectra of (***A.1***) δ, (***A.2***) θ, and (***A.3***) σ bands in NREMS during the light and the dark phases of the 12 h light/dark cycle. NREMS mean power spectra in the 0–25 Hz frequency range during the (***B.1***) light and (***B.2***) dark phases. Power spectra of (***C.1***) δ, (***C.2***) θ, and (***C.3***) σ bands in REMS during the light and the dark phases of the 12 h light/dark cycle. REMS mean power spectra in the 0–25 Hz frequency range during the (***D.1***) light and (***D.2***) dark phases. Vehicle = 10 rats; UCM871 7 mg/kg = 7 rats; UCM871 14 mg/kg = 9 rats; UCM871 21 mg/kg = 7 rats. Data for frequency bands are expressed as mean (percentage of variation vs vehicle) ± SEM. *p < 0.05, **p < 0.01, Vehicle versus UCM871 treatments; ^#^*p *< 0.05, ^##^*p *< 0.01, ^###^*p *< 0.001 light versus dark phase, two-way ANOVA for repeated measures followed by Bonferroni’s post hoc comparisons.

Figure 2 reports the changes induced by UCM871 on the power spectra of REMS and NREMS. During NREMS, UCM871 produced an overall increase of δ (Fig. 2*A*.1; interaction, *F*_3,29 _= 0.916, *p *= 0.445; treatment, *F*_3,29_ = 3.243, *p *= 0.036; phase of the day, *F*_1,29_ = 7.150, *p *= 0.012, two-way ANOVA for repeated measures) and θ (Fig. 2*A*.2; interaction, *F*_3,29 _= 0.129, *p *= 0.941; treatment, *F*_3,29 _= 4.931, *p *= 0.007; phase of the day, *F*_1,29_ = 0.116, *p *= 0.736, two-way ANOVA for repeated measures) power independently from the phase of the day, and did not affect σ power (Fig. 2*A*.3; interaction, *F*_3,29 _= 2.970, *p *= 0.048; treatment, *F*_3,29 _= 2.530; *p *= 0.077; phase of the day, *F*_1,29 _= 19.10; *p *= 0.0001, two-way ANOVA for repeated measures). Power spectra of NREMS after treatment with vehicle and different doses of UCM871 during the light and dark phases are reported in Figure 2, *B*.1 and *B*.2, respectively.

In the spectral analysis of REMS, UCM871 increased of power of δ (Fig. 2*C*.1; interaction, *F*_3,29 _= 2.086, *p *= 0.124; treatment, *F*_3,29 _= 6.313; *p *= 0.0020; phase of the day, *F*_1,29 _= 6.072; *p *= 0.0199, two-way ANOVA for repeated measures), θ (Fig. 2*C*.2; interaction, *F*_3,29 _= 3.532, *p *= 0.027; treatment, *F*_3,29 _= 5.221, *p *= 0.005; phase of the day, *F*_1,29 _= 9.596, *p *= 0.004, two-way ANOVA for repeated measures), and σ (Fig. 2*C*.3; interaction, *F*_3,29 _= 1.083, *p *= 0.3717; treatment, *F*_3,29 _= 3.844, *p *= 0.0197; phase of the day, *F*_1,29 _= 3.471, *p *= 0.0726, two-way ANOVA for repeated measures). Of interest, the treatment–phase of the day interaction was seen only for θ waves, which are the hallmark of REMS. An increase in REM θ power during the dark/active phase in rats treated with 14 mg/kg UCM871 (*p *= 0.0023) was reported (Fig. 2*C*.2).

Power spectra of REMS after treatment with vehicle and different doses of UCM871 during the light and dark phases are reported in Figure 2, *D*.1 and *D*.2, respectively.

### Melatonin MT_1_ receptors are expressed in LC-NE neurons

**Figure 3.**
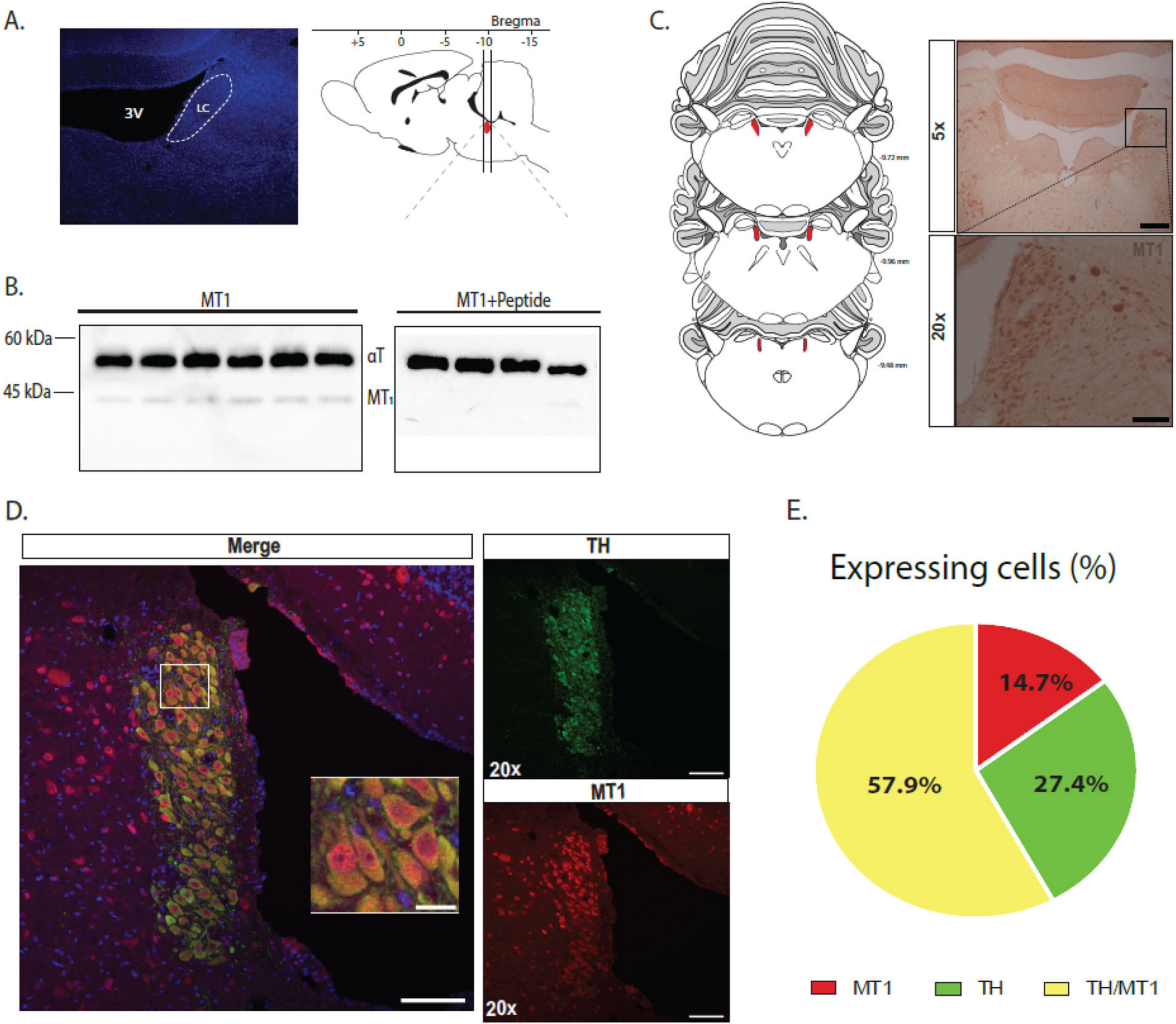
Immunohistochemical expression of the MT_1_ receptor in norepinephrine neurons of the LC. ***A***, Schematical representation of the LC taken for the Western blot analysis. ***B***, Immunoblotting with anti-MT_1_ (1:200) and anti–α-tubulin (aT; 1:10,000) antibodies. Upper bands correspond to the positive blotting from the endogenous control α-tubulin protein whereas the lower bands correspond to the positive blotting from the MT_1_ protein. MT_1_ receptors decorated bands of approximately 40 kDa, at their expected molecular weight. The right panel displays the immunoblot with the immunogen peptide. Each lane represents the blotting from one rat. ***C***, Immunoreactivity of MT_1_ receptors in the LC, 5× (top) and 20× (bottom) show both sides of the LC nucleus expressing MT_1_ receptors. Scale bar: 250 µm (top), 100 µm (bottom). ***D***, Double immunofluorescence confirmed the expression of MT_1_ receptors (red) in LC-NE neurons (TH, green). Sections were counterstained with DAPI (blue). Scale bar: 100 µm, 20 µm (expand). ***E***, Percentage of immunoreactivity of neuronal colocalization (yellow), MT_1_ cellular expression alone (red), and TH neuronal expression alone (green).

The specificity of the MT_1_ antibodies was first assessed using Western blot analyses. In rat LC homogenates, these antibodies revealed a single band in ∼40 kDa (Figure 3*B*), corresponding to the molecular weight of the mammalian MT_1_ receptor (Audinot et al., 2008). Figure 3*A* shows the tissue section corresponding to the LC extracted for the Western blotting technique. No bands were detected with the immunogen peptide (Fig. 3*B*, right panel), suggesting that the protein recognized by the antibodies is the MT_1_ receptor. Then, using these antibodies, we found MT_1_-positive immunostaining at the level of the LC (Fig. 3*C*). Because the MT_1_ receptor was localized in the LC, although it seems less abundant in the ventral part of the LC, we then examined whether the MT_1_ receptors colocalize within NE-positive neurons in the LC, using immunofluorescence. As shown in Figure 3*D*, neurons positive for tyrosine hydroxylase (TH) (green) and MT_1_-positive neurons (red) exhibited high colocalization. Interestingly, while a small population of LC neuronal cells was exclusively immunoreactive with MT_1_ (14.6%) and another one was exclusively immunoreactive with NE positive (27.4%), most LC neurons (57.9%) exhibited a colocalization, showing a TH-like and MT_1_-like immunoreactivity (Fig. 3*E*).

### Acute administration of UCM871 dose-dependently decreases the firing activity of LC-NE neurons in a MT_1_ receptor dependent manner

**Figure 4.**
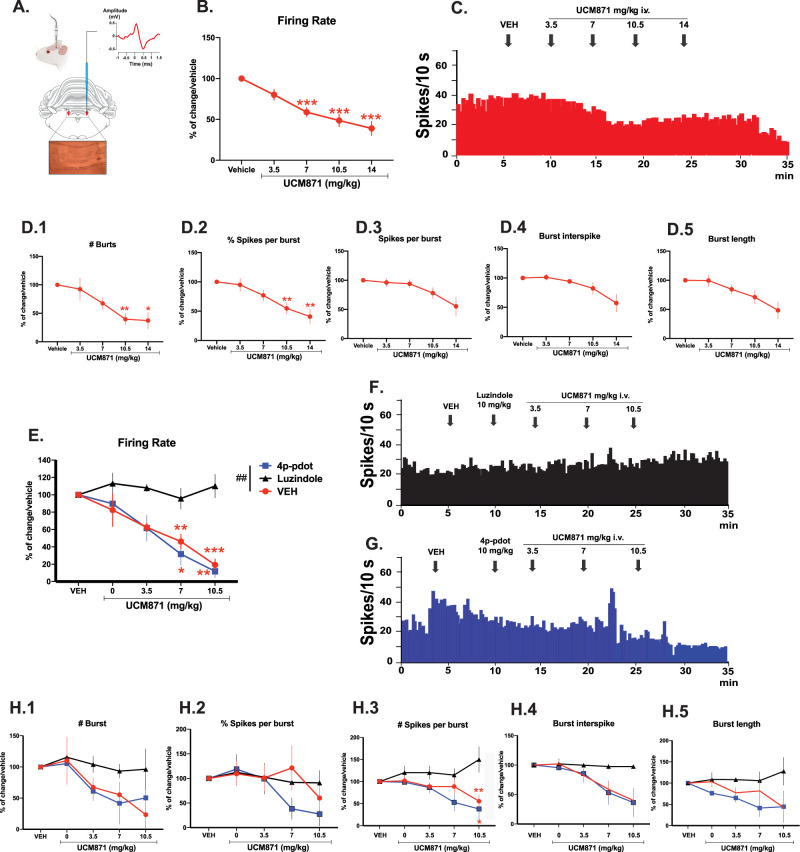
UCM871 decreases the firing activity of locus ceruleus norepinephrine (LC-NE) neurons, and this effect is blocked by the MT_1_/MT_2_ antagonist luzindole, but not by the MT_2_ selective antagonist 4P-PDOT. ***A***, Representative schema and micrograph of a coronal section of the rat brain showing the location of the bilateral LC nucleus (red). Histological control of a recording site in the LC is also shown. Typical spike characteristics of norepinephrine (NE) neurons (top). ***B***, Spontaneous firing rates (% of change vs vehicle injection) of LC-NE neurons after extravascular injection of vehicle and cumulative doses of UCM871 (3.5, 7, 10.5, and 14 mg/kg). The mean ± SEM from 11 neurons (1 neuron per rat) is shown. ***C***, Representative integrated firing rate histogram showing the effects of cumulative doses of UCM871 on the firing rate of LC-NE neurons. ***D.1–5***, Burst-firing activity parameters of LC-NE neurons after extravascular injection of vehicle, and cumulative doses of UCM871 (3.5, 7, 10.5, and 14 mg/kg). Data are reported as mean ± SEM from 11 neurons (1 neuron per rat) are shown. **p < 0.01, ***p < 0.001 versus vehicle, one-way ANOVA for repeated measures followed by Bonferroni’s post hoc comparisons. ***E***, Firing rate of LC-NE neurons pretreated with vehicle (*n* = 9; 1 neuron per rat), 4P-PDOT (*n* = 4; 1 neuron per rat), or luzindole (*n* = 3; 1 neuron per rat) prior to the injection of cumulative doses of UCM871 (3.5, 7, and 10.5 mg/kg). ***F***, Integrated firing rate of LC-NE neurons pretreated with luzindole (top) and 4P-PDOT (bottom) prior to UCM871 administration. ***G*.1–5**, Burst-firing activity parameters of LC-NE neurons pretreated with vehicle (*n* = 9; 1 neuron per rat), 4P-PDOT (*n* = 4; 1 neuron per rat), or luzindole (*n* = 3; 1 neuron per rat) prior to the injection of cumulative doses of UCM871 (3.5, 7, and 10.5 mg/kg). *p < 0.05, **p < 0.01, ***p < 0.001 versus vehicle; ^##^*p *< 0.001 VEH versus UCM871 treatments, two-way ANOVA for repeated measures followed by Bonferroni’s post hoc comparisons.

UCM871 dose-dependently inhibited LC-NE neuronal activity (Fig. 4; *F*_2,29 _= 27.07, *p *< 0.0001, one-way ANOVA for repeated measures), confirming the decrease in LC activity observed in REMS (Osorio-Forero et al., 2022). UCM871 at doses of 7, 10.5, and 14 mg/kg significantly decreased the LC-NE firing rate (*p *= 0.0002, *p* = 0.0005, and *p *= 0.0003, respectively) compared with vehicle (Fig. 4*B,C*). Concerning the burst-firing activity, UCM871 significantly decreased the number of bursts per 200 s (Fig. 4*D*.1; *F*_2,21_ = 8.702, *p *= 0.0013, one-way ANOVA for repeated measures) and the percentage of spikes in bursts (Fig. 4*D*.2; *F*_2,26 _= 10.86, *p *= 0.0001, one-way ANOVA for repeated measures) at the doses of 10.5 (number of bursts per 200 s: *p *= 0.0012; percentage of spikes in bursts: *p *= 0.0094) and 14 mg/kg compared with vehicle (number of bursts per 200 s: *p *= 0.0147; percentage of spikes in bursts: *p *= 0.0091). Although a general effect of UCM871 was observed for the mean spikes per burst (Fig. 4*D*.3; *F*_2,22_ = 5.737, *p *= 0.0078, one-way ANOVA for repeated measures), the mean burst interspikes (Fig. 4*D*.4; *F*_1,17 _= 5.692, *p *= 0.0152, one-way ANOVA for repeated measures), and the mean burst length (Fig. 4*D*.5; *F*_2,21_ = 7.211, *p *= 0.0032, one-way ANOVA for repeated measures), Bonferroni’s post hoc comparisons revealed no significant effect of any tested dose on these electrophysiological parameters.

Next, we pretreated the male rats with either 4P-PDOT (10 mg/kg, *n* = 4, 1 neuron/rat) or luzindole (10 mg/kg, *n* = 3, 1 neuron/rat). We found that pretreatment with luzindole but not with 4P-PDOT prevented the inhibitory effect of UCM871 on the firing activity of LC-NE at the dose of 7 and 10.5 mg/kg compared with vehicle pretreatment (Fig. 4*E–G*; interaction, *F*_8,52 _= 2.53, *p *= 0.02; pretreatment, *F*_2,13 _= 11.08, *p *= 0.001; treatment, *F*_(4. 52)_ = 11.33, *p *< 0.0001, two-way ANOVA for repeated measures), as well as on the number of spikes per burst at the dose of 10.5 mg/kg (Fig. 4*H*.3; interaction, *F*_8,52 _= 2.34, *p *= 0.03; pretreatment, *F*_2,13 _= 7.61, *p *= 0.0065; treatment, *F*_4,52 _= 1.71, *p *= 0.16, two-way ANOVA for repeated measures). No significant effects due to pretreatment with luzindole or 4P-PDOT were found on the number of bursts (Fig. 4*H*.1; interaction, *F*_8,52 _= 0.44, *p *= 0.88; pretreatment, *F*_2,13 _= 0.99, *p *= 0.39; treatment, *F*_4,52 _= 2.55, *p *= 0.05, two-way ANOVA for repeated measures), % of spikes in burst (Fig. 4*H*.2; interaction, *F*_8,52 _= 0.59, *p *= 0.77; pretreatment, *F*_(2.13)_ = 0.39, *p *= 0.68; treatment, *F*_4,52 _= 1.38, *p *= 0.25, two-way ANOVA for repeated measures), burst interspike interval (Fig. 4*H*.4; interaction, *F*_8,52 _= 1.36, *p *= 0.24; pretreatment, *F*_(2.13)_ = 2.39, *p *= 0.13; treatment, *F*_4,52 _= 7.62, *p *< 0.0001, two-way ANOVA for repeated measures) and burst length (Fig. 4*H*.5; interaction, *F*_8,52 _= 0.44, *p *= 0.88; pretreatment, *F*_2,13 _= 0.99, *p *= 0.39; treatment, *F*_4,52 _= 2.55, *p *= 0.05, two-way ANOVA for repeated measures). These data indicate that UCM871 was acting on the LC-NE neurons through an MT_1_ receptor-mediated mechanism, since the MT_1_/MT_2_ antagonist luzindole, but not the MT_2_ antagonist 4P-PDOT, blocked the effects of UCM871.

### In vivo transfection with shRNA by local delivery of an AAV vector targeting MT_1_ receptors in NE-LC neurons

**Figure 5.**
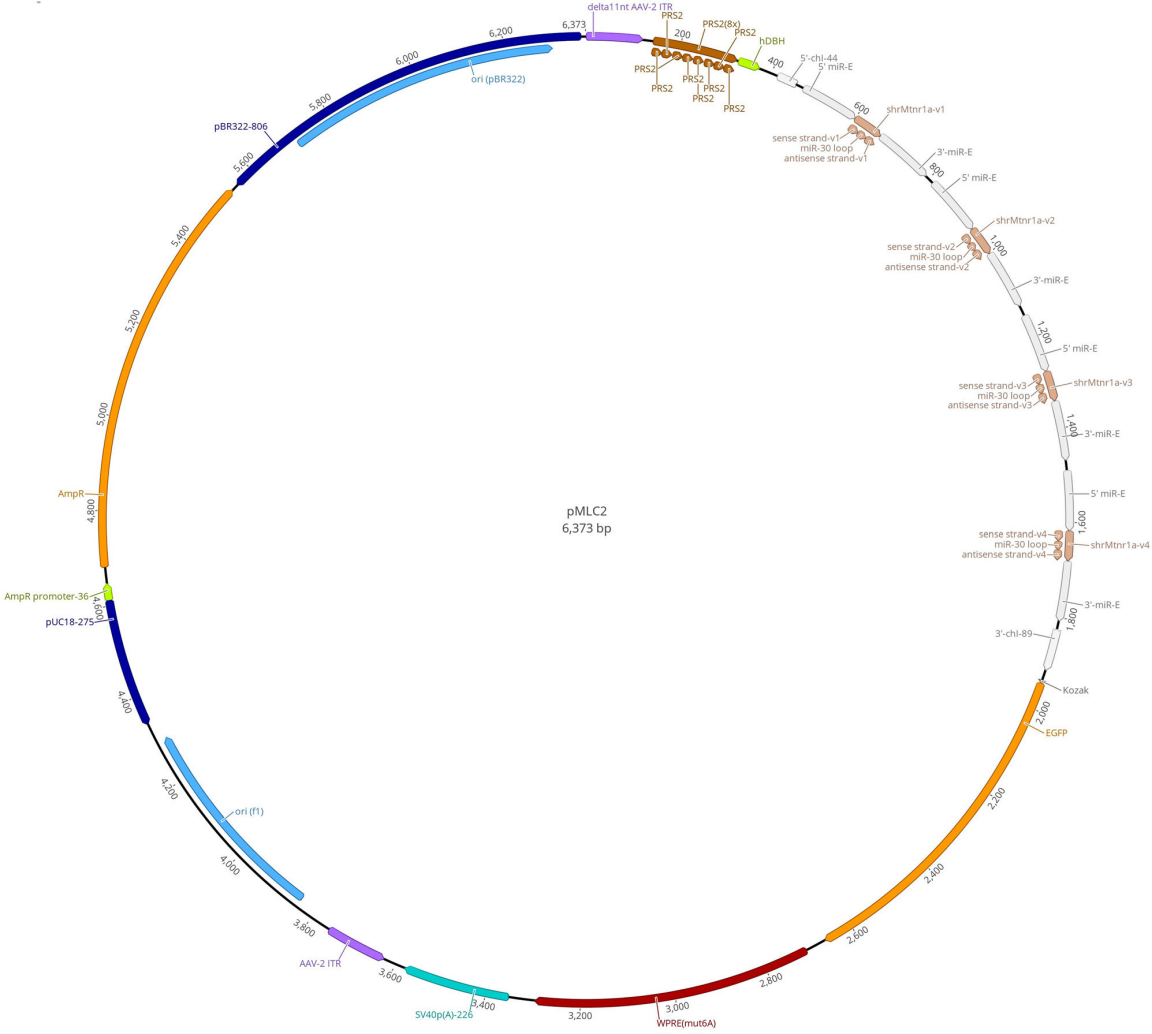
Map of the expression of the AAV vector plasmid pMLC_2_ gene cassette. Each plasmid carries one expression cassette containing two sequences of AAV ITRs from serotype 2 (purple), one hDBH promoter fragment (green) controlled by an 8× promoter (PRS2; brown), which confers selective expression in noradrenergic and adrenergic neurons. The vector contains four sequences coding for Mtr1a (light brown), the EGFP sequence protein (yellow), posttranscriptional regulatory element (WPRE) to enhance expression (red), the reporter gene SV40 promoter (no enhancer) (turquoise), the Class 1 integron ori (blue), the restriction enzyme pUC18-275 (dark blue), and the transcription regulator AmpR promoter-36 (amber).

**Figure 6.**
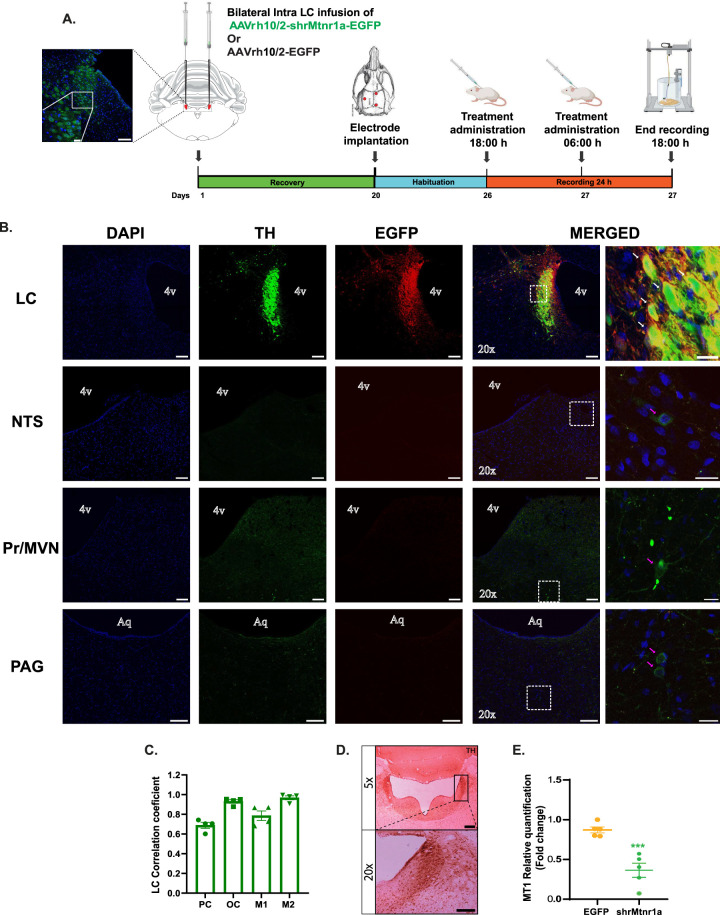
Transduction efficiency of the AAV-hrMT_1_ viral vector is detectable in LC-NE TH^+^ neurons but negligible in nucleus tractus solitarius (NTS), nucleus prepositus/medial vestibular nucleus (Pr/MVN), and periaqueductal grey (PAG) TH^+^ neurons. ***A***, Schematic timeline of the experiments and representative schema of coronal sections of the rat brain viewing the bilateral injection site (right) and histological section (left), showing the fluorescence of cell nuclei DAPI (blue) and endogenous tyrosine hydroxylase (TH, green) immunoreactivity. Scale bars: 100 and 20 µm in expand. ***B***, Viral vector (shrMT_1_) transduction after bilateral injection of 1 µl shrMT_1_ in the LC was analyzed by confocal microscopy of coronal sections. Each row represents a different tissue section adjacent to the third ventricle, locus coeruleus (LC), nucleus tractus solitarius (NTS), nucleus prepositus/medial vestibular nucleus (Pr/MVN), and periaqueductal grey (PAG). The first column represents a cell nuclei DAPI (blue) fluorescence; the second column shows endogenous TH (green) immunoreactivity; the third column shows the shrMtnr1a transduction (GFP; red) immunoreactivity; the fourth column shows TH/GFP coexpression leveling cells; and the last column shows the magnification of the TH/GFP coexpression labeling cells. Structures that are positive for TH are indicated with a pink arrow, and structures positive for both TH/GFP are indicated with a white arrow. Scale bars: 100 µm in ***B*** Columns 1, 2, 3, and 4; and 20 µm in ***B*** Column 4. ***C***, Thresholds for the images collected in ***B*** were acquired using the Auto Threshold ImageJ plugin (Landini), and Pearson's and Mander's coefficients were calculated with the JACoP plugin on the average of four animals. PC, Pearson’s correlation; OC, overlap correlation; M1, Mander’s overlap EGFP; M2, Mander’s overlap TH. ***D***, Immunoreactivity of TH in the LC, 5× (top) and 20× (bottom) after bilateral administration of 1 µl shrMT_1_ confirming the integrity of the norepinephrine-expressing cells in the LC. Scale bars: 250 µm in ***C*** top; 100 µm in ***C*** bottom. ***E***, Fold change detection in LC nucleus of animals transduced with virus expression control (EGFP; *n* = 5 rats) or the shRNA specific (shrMT_1_; *n* = 5 rats). Data are reported as mean ± SEM. ***p < 0.001, Student’s's *t* test (unpaired analysis).

We constructed an adeno-associated virus (AAV) vector serotype 10/2 encoding the EGFP and expressing four different shrMT_1_ RNAs under the control of the artificial promoter PRS (8×) with hDBH promoter (Fig. 5) to selectively target and silence the expression of MT_1_ receptors only in NE neurons. We then injected the vector AAVrh10/2-shrMT_1_-EGFP or the vector AAVrh10/2-EGFP as control bilaterally into the LC of adult rats. The specificity of expression was evaluated 4 weeks postinjection (Fig. 6*A*), since AAV vectors have been shown to mediate robust transgene expression in most tissues (Hordeaux et al., 2015). To assess the in vivo transfection efficiency of mRNA by AAV vectors, we evaluated positive transfection by immunofluorescence (anti-GFP) in LC-NE neurons and by qRT-PCR. To visualize the expression of the AAV-hrMT_1_ viral vector, 26 d after injection, we analyzed the transgene expression by confocal microscopy of coronal sections which were immunostained against TH (to visualize endogenous noradrenergic neurons in the LC) and EGFP (to enhance the signal originating from AAV expression). Nuclei adjacent to the LC and to the 4th ventricle were also stained to rule out possible spilling of the virus from the injection site (Fig. 6*B*). In the solitary tractus (NST), the prepositus nucleus/mediovestibular nucleus magnocellular (Pr/MVeMC), and the periaqueductal area (PAG), only a few TH^+^ neurons were found, in keeping with the paucity of NE soma in non-LC areas (Svensson, 1987; Berridge and Waterhouse, 2003). A colabeling was observed in only very few neurons, mostly in the area corresponding to the mesencephalic trigeminal nucleus, which contains also TH^+^ neurons. On the contrary, in all animals (*n* = 4), clear vMLC2-rh10 (EGFP = RED) transduction was present in the soma of NE-positive neurons within the LC (Fig. 6*B*). Next, we used these images for colocalization analyses applying Auto Threshold ImageJ plugin (Landini) and JACoP plugin option of the image analysis software Fiji. High correlation values (Fig. 6*C*) were found by Pearson's (PC; 0.68 ± 0.03) and overlap colocalization (OC; 0.93 ± 0.017). To evaluate the efficacy of the viral transduction, defined as the fraction of TH^+^ cells coexpressing EGFP over the total number of TH^+^ cells (Mander's coefficient M2), we compared the overlap of pixels between the pixels count of TH^+^ cells with the pixels of EGFP^+^ cells and found an overlap of ≥96% of the TH^+^ cells as positively transduced. Similarly, we quantified the specificity (Mander's coefficient M1) of transgene (AAV-hrMT_1_) expression as the fraction of EGFP^+^ cells that coexpress TH^+^ cells. Thereby, we found that ≥78% of the EGFP^+^ cells are positively transduced in TH^+^ cells (Fig. 6*C*). No damage to the LC given by the cannulation as well as the injection was observed (Fig. 6*D*).

Quantification of MT_1_ receptors by qRT-PCR was performed in LC of rats injected with either the AAVrh10/2-hr MT_1_-EGFP or the control AAVrh10/2-EGFP. A significant decrease in MT_1_ mRNA levels was detected in LC (AAVrh10/2-hrMT_1_-EGFP = 0.3648-fold changes vs AAVrh10/2-EGFP = 0.872-fold changes; *t*_8 _= 5.263, *p *= 0.0008, unpaired *t* test) of AAVrh10/2-hrMT_1_-EGFP compared with control AAVrh10/2-EGFP rats, demonstrating the transfection efficiency and specificity of shRNA in the LC area (Fig. 6*E*).

**Figure 7.**
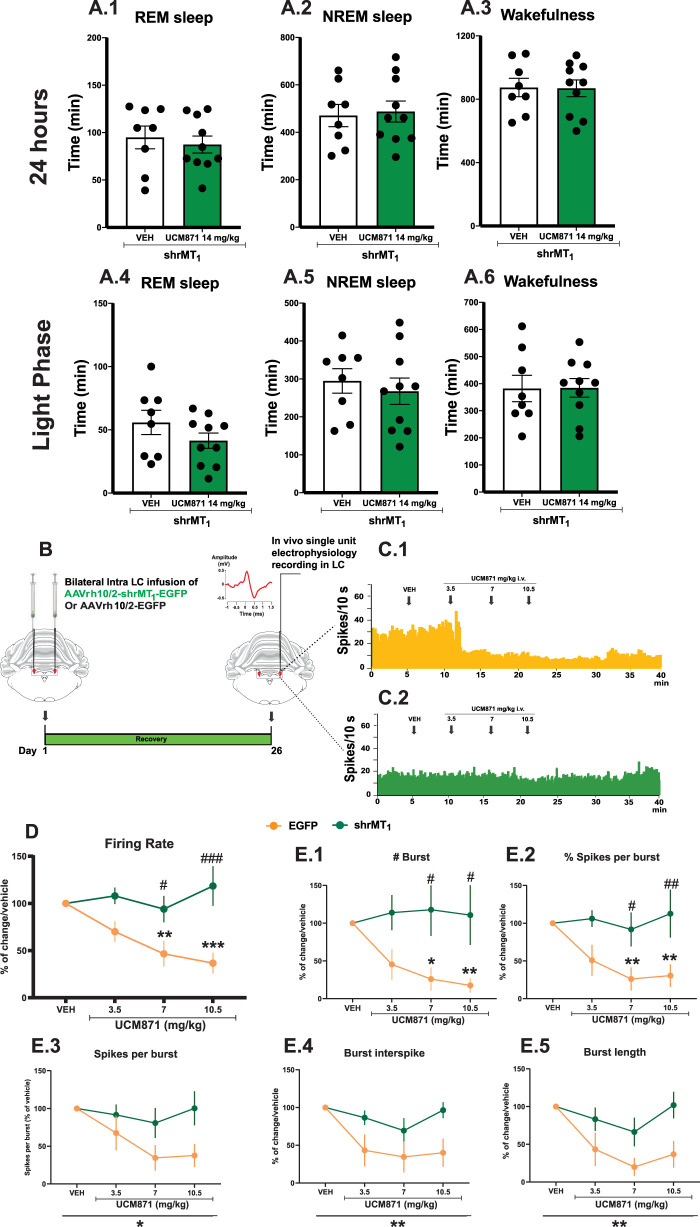
The knockdown of melatonin MT_1_ receptors in LC-NE neurons abolishes the effects of UCM871 on REM sleep and on LC-NE firing activity. ***A***, Cumulative analysis of the 24 h duration of the three vigilance states REMS (***A.1***), NREMS (***A.2***), and wakefulness (***A.3***) of transfected animals after the administration of UCM871 at 14 mg/kg (*n* = 10 rats) or vehicle (*n* = 8 rats). Duration of REMS (***A.4***), NREMS (***A.5***), or wakefulness (***A.6***) during the inactive/light phase in animals transfected with shMT_1_ and treated with vehicle (*n* = 8 rats) or UCM871 at 14 mg/kg (*n* = 10 rats). ***B***, Schematic timeline of the electrophysiological experiment. ***C***, Example of spontaneous firing rate histograms of LC-NE neurons after treatment with cumulative doses of UCM871 in rats transfected with control (yellow) and AAV-shMT_1_ (green). ***D***, Spontaneous firing rate of LC-NE neurons after administration of cumulative doses of UCM871 (3.5, 7, and 10.5 mg/kg) in rats transfected with control (yellow; *n* = 6, 1 neuron/rat) and AAV-shMT_1_ (green; *n* = 6, 1 neuron/rat). ***E.1–5***, Burst-firing activity parameters of LC-NE neurons transfected with control (yellow; *n* = 6, 1 neuron/rat) and AAV-shMT_1_ (green; *n* = 6, 1 neuron/rat) following cumulative doses of UCM871. *p < 0.05, **p < 0.01, ***p < 0.001 versus vehicle; ^#^*p *< 0.05, ^##^*p *<0.01, ^###^*p *< 0.001, shMT_1_ versus EGFP, by two-way ANOVA for repeated measures followed by Bonferroni’s post hoc test.

UCM871 (14 mg/kg) did not affect the duration of REMS (Fig. 7*A*.1; *t*_16 _= 0.5157, *p *= 0.61, unpaired *t* test), NREMS (Fig. 7*A*.2; *t*_16 _= 0.2625, *p *= 0.80, unpaired *t* test), and wakefulness (Fig. 7*A*.3; *t*_16 _= 0.06505, *p *= 0.95, unpaired *t* test) over 24 h in animals infused with AAVrh10/2-shrMT_1_a-EGFP.

Given that the effects of UCM871 on vigilance states are mostly due to changes occurring during the light/inactive phase in naive animals (Fig. 1*D*), we examined the effect of UCM871 (14 mg/kg) in animals infused with AAVrh10/2-hr MT_1_-EGFP during the light/inactive phase. Interestingly, UCM871 had no effect on the duration of REMS (Fig. 7*A*.4; *t*_16 _= 1.313, *p *= 0.21, unpaired *t* test), NREMS (Fig. 7*A*.5; *t*_16 _= 0.5602, *p *= 0.58, unpaired *t* test), and wakefulness (Fig. 7*A*.6; *t*_16 _= 0.04086, *p *= 0.971, unpaired *t* test) in AAVrh10/2-hr MT_1_-EGFP rats during the light/inactive phase compared with vehicle-treated animals. These results demonstrate that the effect of UCM871 (14 mg/kg) on REMS requires an intact expression of MT_1_ receptors in LC-NE neurons.

**Figure 8.**
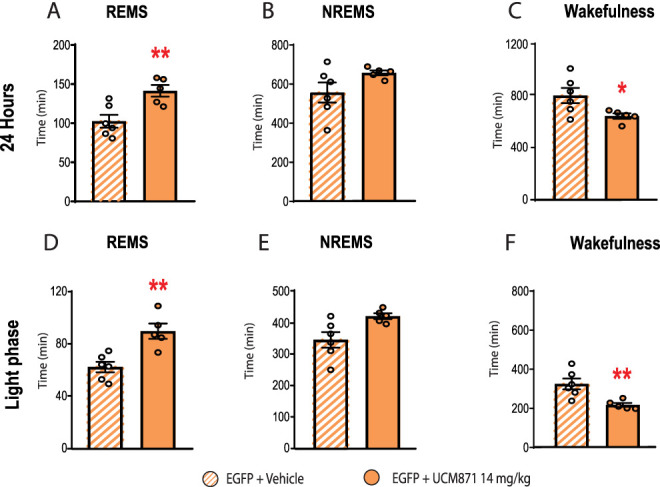
Effects of the administration of UCM871 (14 mg/kg) on the sleep/wake cycle of rats injected with the viral expression control protein (enhanced green fluorescent protein, EGFP). UCM871 (14 mg/kg) increases the duration of (***A***) REMS but not of (***B***) NREMS and decreases the duration of (***C***) wakefulness over 24 h. Similar effects of UCM871 (14 mg/kg) on (***D***) REMS, (***E***) NREMS, and (***F***) wakefulness are present also during the light phase. EGFP + vehicle = 6 rats; EGFP + UCM871 14 mg/kg = 5 rats. Data are expressed as mean ± SEM. *p < 0.05, **p < 0.01, Student’s *t* test.

As a positive control, we tested whether UCM871 administration induced changes in the sleep/wake cycle of rats infused with the control viral vector (AAVrh10/2-EGFP). As shown in Figure 8*A*, we found a significant increase in REMS duration during the 24 h (*t*_9 _= 3.430, *p *= 0.0075, unpaired *t* test) in animals treated with UCM871 (14 mg/kg) compared with those receiving vehicle. The 24 h duration of NREMS (Fig. 8*B*; *t*_9 _= 1.549, *p *= 0.1558, unpaired *t* test) was not affected by UCM871 (14 mg/kg), while wakefulness over 24 h (Fig. 8*C*) was shorter in AAVrh10/2-EGFP rats treated with UCM871 (14 mg/kg) than with vehicle (*t*_9 _= 2.37, *p *= 0.0414, unpaired *t* test). During the light/inactive phase, UCM871 (14 mg/kg) increased the duration of REMS (Fig. 8*D*; *t*_9 _= 2.074, *p *= 0.0302, unpaired *t* test), decreased the duration of wakefulness (Fig. 8*F*; *t*_9 _= 3.346, *p *= 0.0086, unpaired *t* test), but did not affect the duration of NREMS (Fig. 8*E*; *t*_9 _= 2.074, *p *= 0.0679, unpaired *t* test) in AAVrh10/2-EGFP rats. Taken together, these results indicate that infection with the control AAVrh10/2-EGFP viral vector neither affected the physiological sleep/wake cycle nor the effects of UCM871 (14 mg/kg) on the vigilance states, as they mirror those obtained in naive animals (Fig. 1).

### The knockdown of melatonin MT_1_ receptors in LC-NE neurons suppresses the effects of UCM871 on the firing activity of LC-NE neurons

Finally, we tested the cumulative doses of UCM871 (3.5, 7 and 10.5 mg/kg, i.v.) over LC-NE neuronal activity in control AAVrh10/2-EGFP (*n* = 6 neurons with 1 neuron/rat, Fig. 7*C*.1) or AAVrh10/2-hr MT_1_-EGFP infused animals (*n* = 6 neurons with 1 neuron/rat, Fig. 7*C*.2). Rats infused with AAVrh10/2-EGFP (control) and treated with UCM871 at doses of 7 mg/kg (*p *= 0.001) and 10.5 mg/kg (*p *= 0.0003) had a significant decrease in LC-NE firing activity in comparison with vehicle (Fig. 7*C*.1,*D*). On the contrary, cumulative doses of UCM871 failed to decrease LC-NE neural activity in AAVrh10/2-hr MT_1_-EGFP rats (with MT_1_ knockdown) (Fig. 7*C*.2,*D*; interaction, *F*_3,30 _= 7.436, *p *= 0.0007; MT_1_ knockdown, *F*_1,10 _= 13.32, *p *= 0.004; treatment, *F*_3,30 _= 4.484, *p *= 0.01, two-way ANOVA for repeated measures).

Moreover, MT_1_ knockdown also blocked the changes induced by UCM871 on the different parameters of LC-NE burst-firing activity (Fig. 7*E*1–5): number of bursts (interaction, *F*_3,30 _= 3.52, *p *= 0.026; MT_1_ knockdown, *F*_1,10 _= 5.58, *p *= 0.039; treatment, *F*_3,10 _= 1.75, *p *= 0.177); % of spikes in burst (interaction, *F*_3,30 _= 3.25, *p *= 0.035; MT_1_ knockdown, *F*_1,10 _= 7.705, *p *= 0.0196; treatment, *F*_3,30 _= 3.03, *p *= 0.0442); spikes per burst (interaction, *F*_3,30 _= 1.819, *p *= 0.165; MT_1_ knockdown, *F*_1,10 _= 4.81, *p *= 0.052; treatment, *F*_3,30 _= 3.199, *p *= 0.037); burst interspike (interaction, *F*_3,30 _= 2.059, *p *= 0.126; MT_1_ knockdown, *F*_1,10 _= 5.397; *p *= 0.042; treatment, *F*_3,30 _= 0.888, *p *= 0.002); and burst length (interaction, *F*_3,30 _= 1.825, *p *= 0.163; MT_1_ knockdown, *F*_1,10 _= 4.438, *p *= 0.011, treatment, *F*_3,30 _= 5.377, *p *= 0.004).

## Discussion

In this study, we investigated the effects of the selective MT_1_ receptors partial agonist UCM871 on the vigilance states in rats, and we elucidated its underlying mechanism of action. UCM871, by selectively activating the MT_1_ receptors, increased the duration of REMS during the 24 h light/dark cycle, mostly because of the effect of the drug on REMS during the light/inactive phase. Notably, we showed that the promotion of REMS is likely the result of the activation of MT_1_ receptors localized in LC-NE neurons since (1) UCM871 inhibits LC-NE neuronal activity in a dose-dependent manner and through an MT_1_-mediated mechanism as its activity is blocked by the non-selective MT_1_/MT_2_ receptor antagonist luzindole, but not by the selective MT_2_ receptor antagonist 4P-PDOT; (2) the knockdown of melatonin MT_1_ receptors in LC-NE neurons by a novel selective AAV vector nullifies the effect of UCM871 on REMS duration; and (3) no inhibitory effects of UCM871 on LC-NE neurons are present in rats with knocked-down MT_1_ receptors in LC-NE neurons.

These data are consistent with our initial hypothesis based on previous results in MT_1_ receptors knock-out mice. In those mice, we observed a significant reduction in REMS duration over the 24-hour sleep/wake cycle, primarily attributable to decreased REMS during the light/inactive phase (Comai et al., 2013). The LC is the primary source of NE projections throughout the central nervous system (Aston-Jones and Waterhouse, 2016) and, as part of the ascending arousal pathway, is involved in the maintenance of the arousal state (Aston-Jones and Bloom, 1981; Ross and Van Bockstaele, 2021). Physiologically, the rate of spontaneous discharge of LC-NE neurons varies among the different vigilant stages, with higher activity during wakefulness, lower activity during NREMS, and virtually no activity during REMS (Aston-Jones and Bloom, 1981; Fuller et al., 2006; Aston-Jones and Waterhouse, 2016). Notably, we found that LC-NE neurons express MT_1_ receptors in keeping with the data in mice by Klosen et al. (2019) showing scattered lightly labeled MT_1_-LacZ cells in the LC, and that the MT_1_ receptor partial agonist UCM871 dose-dependently inhibits the activity of LC-NE neurons. Carter et al. (2010) demonstrated that while the optogenetic inactivation of LC-NE neurons in mice resulted in a decrease in both the overall wakefulness and the duration of individual awake episodes, optogenetic activation of LC-NE neurons instead induced immediate sleep-to-wake transitions, further implicating a causal role of LC in maintaining arousal. Interestingly, we reported that selective knockdown of MT_1_ receptors in LC-NE neurons did not affect the duration of the three vigilance states, in agreement with previous studies showing no significant changes in the duration of vigilance states in animals where the NE synthesis was genetically inhibited (Hunsley and Palmiter, 2003) or the LC was pharmacologically lesioned (Lidbrink, 1974; Jones et al., 1977; Blanco-Centurion et al., 2007). Collectively, our data demonstrate that the activation of MT_1_ receptors located in LC-NE neurons is sufficient for inducing REMS. However, since MT_1_ receptors are also present in other brain regions implicated in sleep and projecting to the LC, such as the dorsal raphe nucleus, the suprachiasmatic nucleus (SCN), and the lateral hypothalamus (Lacoste et al., 2015; Sharma et al., 2018; Comai et al., 2019), they could be indirectly participating in the regulation of REMS. Indeed, the regulation of circadian activity of the LC comes from indirect projections from the SCN via the dorsomedial nucleus and paraventricular nucleus of the hypothalamus, as well as medial and ventrolateral preoptic areas (Aston-Jones et al., 2001; Fuller et al., 2006). Given that we administered UCM871 systemically, we cannot rule out the contribution of these or other areas of the brain to the observed effects of UCM871 on sleep and LC-NE activity.

Our group and others have previously demonstrated that selective activation of MT_2_ receptors significantly promotes NREMS (Fisher and Sugden, 2009; Ochoa-Sanchez et al., 2011, 2014), while knocking out MT_2_ receptors in mice significantly reduced the duration of NREMS (Comai et al., 2013). Collectively, these novel results further support the idea that the two melatonin receptors may modulate complementary or even opposite effects on sleep regulation (Gobbi and Comai, 2019a,b) and are a promising pharmacological target for developing new drugs (Liu et al., 2016). In keeping with this, nonselective MT_1_/MT_2_ receptor agonists including melatonin itself or UCM793 have negligible effects on REMS or NREMS (Gobbi and Comai, 2019a,b). These data confirm the need to be selective rather than nonselective when targeting the melatonin system in neuropsychopharmacology.

This study shows that it is possible to activate REMS through a selective MT_1_ receptor agonism in keeping with the role of the melatonin system in REMS. Indeed, according to Wang et al. (2011), melatonin in combination with clonazepam is the only medication indicated for REM sleep behavior disorder. Moreover, melatonin plays an important role in the impaired inhibitory neurotransmission, which is involved in the pathogenesis of REMS behavior disorder (Brooks and Peever, 2011).

It is important to mention that while activation of MT_2_ receptors leads to a significant increase in NREMS paralleled by an increase in the power of δ band (Ochoa-Sanchez et al., 2011, 2014), activation of MT_1_ receptors with UCM871 induces a significant increase of REMS paralleled by an increase in the power of θ band, which is a hallmark of REMS. Moreover, the dose-dependent effects of UCM871 on the duration of REMS were also paralleled by an increase of δ and σ power in REMS but also an increase in δ and θ power in NREMS indicating that UCM871 may enhance the depth or intensity of sleep. Of interest, we found that the increase in the duration of REMS induced by UCM871 occurred without affecting significantly the overall architecture of sleep (bouts of REMS and NREMS as well as sleep fragmentation), as we observed only a minimal—but nonsignificant—increase in the number and duration of REMS bouts. This unique effect on the neural activity patterns and REMS dynamics deserves to be further investigated to understand their potential implications in sleep regulation and pharmacological application.

REMS has an important function in the mammalian brain since it mediates spatial and contextual memory consolidation (Boyce et al., 2017), in part due to the sparse activity of hippocampal adult-born neurons (Kumar et al., 2020) and activity- and experience-dependent synaptic plasticity as well as experience-dependent synapse elimination (Zhou et al., 2020). Interestingly, both NREMS and REMS stages seem to play a complementary role in the process of learning (Tamaki et al., 2020), and their “flip-flop switch” is essential for human physiology (Lu et al., 2006). However, the mechanisms underlying the switch between NREMS and REMS stages remain largely unknown. In this context, we cannot rule out a role played by MT_1_ and MT_2_ receptors in the “flip-flop switch” between REM and NREM; indeed, they are differently expressed during the light/inactive phase (Pinato et al., 2017) and expressed in different brain regions modulating the sleep/wake cycle (Gobbi and Comai, 2019a,b). According to the “flip-flop switch” model, when LC-NE neurons become active, they inhibit the NREMS-promoting neurons, effectively “flipping the switch” from NREMS to wakefulness. Conversely, when they are less active, they allow REMS-promoting neurons to become more active (Lu et al., 2006; Dunmyre et al., 2014). Within this framework, serotonergic neurons of the dorsal raphe also inhibit NREMS-promoting neurons. When these 5-HT neurons become almost silent, they facilitate the activation of REMS-promoting neurons (Lu et al., 2006).

Interestingly, during REM sleep, the serotonin neurons of the dorsal raphe nucleus decrease their activity (Jones, 2005); in keeping, preliminary data showed that UCM871 acutely inhibits serotonergic neurons of the dorsal raphe nucleus (Comai et al., 2017). Consequently, a possible contribution of the dorsal raphe, which expresses MT_1_ receptors (Lacoste et al., 2015), cannot be ruled out.

This study has some limitations: UCM871 was not tested in animals with impaired sleep and/or psychiatric disorders with comorbid sleep impairment or was only partially tested in female rats, where it decreased the LC-NE activity in a dose-dependent manner similar to males (unpublished data). Addressing these drawbacks will help move MT_1_ compounds toward a possible clinical trial in humans, after toxicological studies. Despite these limitations, the present research demonstrates that selective activation of melatonin MT_1_ receptors by UCM871 promotes REMS through inhibition of LC-NE neurons that express MT_1_ receptors. These results add important novel insights to our current knowledge of the neurobiology of sleep. More importantly, they suggest that the MT_1_ receptor may represent a novel selective pharmacological target for drug discovery in diseases characterized by REMS loss and impairments. Remarkably, REM Sleep Behavior Disorder (RBD) has been estimated to affect approximately 0.5–1% of the general population, representing a serious risk factor for the development of neurodegenerative disorders, including Parkinson's disease and other related conditions (Högl et al., 2022). While the precise role of MT_1_ receptors in Parkinson's disease is still under investigation, some studies have suggested that melatonin via MT_1_ receptors may exert neuroprotective effects while modulating circadian rhythms (Comai et al., 2019). Moreover, the lack of MT_1_ receptors in transgenic mice not only impairs REMS, but generates a melancholic-like phenotype (Comai et al., 2015), and activation of MT_1_ receptors improves a manic-like phenotype in mice (Tassan Mazzocco et al., 2023). Future research should address whether dysfunction of the MT_1_ receptor may represent a common mechanism linking sleep, psychiatric disorders, and neurodegenerative diseases.

## Data Availability

All data associated with this study are available upon a reasonable request to the corresponding authors (G.G., gabriella.gobbi@mcgill.ca; S.C., stefano.comai@unipd.it). A material transfer agreement will be required for the sharing of materials.
